# Lentzea sokolovensis sp. nov., Lentzea kristufekii sp. nov. and Lentzea miocenica sp. nov., rare actinobacteria from Miocene lacustrine sediment of the Sokolov Coal Basin, Czech Republic

**DOI:** 10.1099/ijsem.0.006335

**Published:** 2024-04-17

**Authors:** Ana Catalina Lara, Lucie Kotrbová, Moritz Keller, Imen Nouioui, Meina Neumann-Schaal, Yvonne Mast, Alica Chroňáková

**Affiliations:** 1 Biology Centre Czech Academy of Sciences, Institute of Soil Biology and BiogeochemistryNaSádkách 7, 37005 České Budějovice, Czech Republic; 2 University of Chemistry, and Technology, Prague, Faculty of Food and Biochemical Technology, Department of Biochemistry and Microbiology, Technická 5, 16628 Prague, Czech Republic; 3 University of South Bohemia, Faculty of Science, Branišovská 31, 37005 České Budějovice, Czech Republic; 4 Leibniz Institute DSMZ - German Collection of Microorganisms and Cell Cultures, Inhoffenstraße 7B, 38124 Braunschweig, Germany

**Keywords:** Actinomycetes, *Lentzea*, polyphasic taxonomy, post-mining sites

## Abstract

The taxonomic position of three actinobacterial strains, BCCO 10_0061^T^, BCCO 10_0798^T^, and BCCO 10_0856^T^, recovered from bare soil in the Sokolov Coal Basin, Czech Republic, was established using a polyphasic approach. The multilocus sequence analysis based on 100 single-copy genes positioned BCCO 10_0061^T^ in the same cluster as *Lentzea waywayandensis*, strain BCCO 10_0798^T^ in the same cluster as *Lentzea flaviverrucosa*, *Lentzea californiensis*, *Lentzea violacea*, and *Lentzea albidocapillata*, and strain BCCO 10_0856^T^ clustered together with *Lentzea kentuckyensis* and *Lentzea alba*. Morphological and chemotaxonomic characteristics of these strains support their assignment to the genus *Lentzea*. In all three strains, MK-9(H_4_) accounted for more than 80 % of the isoprenoid quinone. The diagnostic diamino acid in the cell-wall peptidoglycan was *meso*-diaminopimelic acid. The whole-cell sugars were rhamnose, ribose, mannose, glucose, and galactose. The major fatty acids (>10 %) were iso-C_15 : 0_, anteiso-C_15 : 0_, iso-C_16 : 0_, and C_16 : 0_. The polar lipids were diphosphatidylglycerol, methyl-phosphatidylethanolamine, phosphatidylethanolamine, hydroxy-phosphatidylethanolamine, phosphatidylglycerol, and phosphatidylinositol. The genomic DNA G+C content of strains (mol%) was 68.8 for BCCO 10_0061^T^, 69.2 for BCCO 10_0798^T^, and 68.5 for BCCO 10_0856^T^. The combination of digital DNA–DNA hybridization results, average nucleotide identity values and phenotypic characteristics of BCCO 10_0061^T^, BCCO 10_0798^T^, and BCCO 10_0856^T^ distinguishes them from their closely related strains. Bioinformatic analysis of the genome sequences of the strains revealed several biosynthetic gene clusters (BGCs) with identities >50 % to already known clusters, including BGCs for geosmin, coelichelin, ε-poly-l-lysine, and erythromycin-like BGCs. Most of the identified BGCs showed low similarity to known BGCs (<50 %) suggesting their genetic potential for the biosynthesis of novel secondary metabolites. Based on the above results, each strain represents a novel species of the genus *Lentzea*, for which we propose the name *Lentzea sokolovensis* sp. nov. for BCCO 10_0061^T^ (=DSM 116175^T^), *Lentzea kristufekii* sp. nov. for BCCO 10_0798^T^ (=DSM 116176^T^), and *Lentzea miocenica* sp. nov. for BCCO 10_0856^T^ (=DSM 116177^T^).

## Data Summary

Seven supplementary tables and three supplementary figures are available with the online Supplementary Material.

## Introduction

The genus *Lentzea* (family *Pseudonocardiaceae*, order *Pseudonocardiales*) was first proposed in 1995 [1]. The type species *Lentzea albidocapillata* IMMIB D-958^T^ (=DSM 44073^T^) was isolated from an abdominal mass of a patient suffering from peritoneal carcinomatosis and colon carcinoma [1]. Currently, the genus encompasses 27 validly described species [2] and two subspecies (May 2023; https://lpsn.dsmz.de/genus/lentzea), which have been isolated from diverse sources, including human pathological tissue, equine placenta, karst caves, plant, soils of various types (desert soil, wheat field soil, acid soil, undefined soils, rhizosphere, and Tibetan plateau soil), and 10 strains of unknown sources. Members of this genus are Gram-positive, non-motile, mostly aerobic actinobacteria with branched aerial mycelia that fragments into rod-shaped elements [1]. The aerial hyphae are white to whitish-yellow; sporangia are not produced. The genus is characterized by: the presence of *meso*-diaminopimelic acid (*meso*-DAP) in the cell walls (cell wall chemotype III); cell membranes with fatty acids that can be branched, straight saturated or unsaturated chains (phospholipid type II); MK-9 (H_4_) as the most abundant menaquinone; phosphatidylethanolamine, diphosphatidylglycerol, phosphatidylglycerol, and phosphatidylinositol as the most abundant polar lipids; and whole-cell lysates with galactose, mannose, and ribose as predominant sugars [1].

The genus *Lentzea* has recently attracted a great deal interest due to its underestimated genetic diversity [3,4], including its genetic potential for biosynthesis of natural products that are in high demand in the pharmaceutical field [5]. For instance, lentzeosides with anti-HIV-1 integrase activity were isolated from the extremotolerant *Lentzea* sp. strain H45 [6]. Later, petrichorins A and B, produced by *Lentzea flaviverrucosa* DSM 44664^T^, were shown to exhibit antitumor activity *in vitro* [7]. In general, the genus *Lentzea* shows the third highest biosynthetic novelty index among rare actinobacteria genera [4], suggesting that isolates of *Lentzea* sp. from various oligotrophic or extreme environments could be a good source of novel natural products. However, to date (May 2023), only 21 good-quality genomes of the 27 species validly published under the International Code of Nomenclature of Prokaryotes (ICNP) are publicly available, with the reference genomes of *Lentzea deserti* and *Lentzea atacamensis* being identical [8], and the sole exception of *Lentzea indica*, which is not validly published under the ICNP but has a very complete genome in comparison with the other strains [5].

Strains BCCO 10_0061^T^, BCCO 10_0798^T^, and BCCO 10_0856^T^ were isolated from Miocene lacustrine clay material (pH 7.8) deposited as overburden after brown coal mining (for more details on the geology, see Kříbek *et al*. [9]), as part of a diversity study and search for genes encoding unique biosynthetic enzymes [10]. The overburden material was heaped in ridges with depressions, and the sites on the locality were left to undergo primary succession. All three strains were isolated from early successional plots, 10–15 years after heaping [11]. The aim of this study was to determine the taxonomic status of the three new isolates using a genomic and polyphasic approach.

## Isolation and ecology

All strains were isolated from soil at the top of reclaimed mine heaps in the early successional stage in the Sokolov brown coal mine district area in the Czech Republic. Strains were isolated from bare soil developed on Miocene lacustrine clay deposited after brown coal mining in 2004 (BCCO 10_0061^T^) on McBeth Scales agar (for details see Chroňáková *et al.* [11]), in 2007 (BCCO 10_0798^T^), and in 2008 (BCCO 10_0856^T^) on Reasoner's 2A (R2A) agar (HiMedia). Cultures were maintained on yeast extract–malt extract agar (International *Streptomyces* Project (ISP) 2) [12] and as cell suspensions in 15 % (v/v) glycerol stocks at −76 °C for long-term preservation. Strains were deposited in the Culture Collection of Actinomycetes of the Biology Centre Collection of Organisms (https://actinomycetes.bcco.cz/) and at the Leibniz Institute DSMZ – German Collection of Microorganisms and Cell Cultures GmbH (www.dsmz.de/).

## 16S rRNA gene analysis

Genomic DNA was isolated from 3-day-old cultures grown aerobically (150 r.p.m.) in Luria–Bertani medium broth [13] using the NucleoSpin Microbial DNA Kit (Macherey-Nagel) according to manufacturer’s recommendations. Quantity and quality of the genomic DNA was analysed by a NanoDrop 2000 spectrophotometer (Thermo Scientific) and by agarose gel electrophoresis. The complete 16S rRNA gene was amplified by PCR using the bacterial universal primers pA and pH [14]. The purified PCR products were bidirectionally sequenced in a commercial laboratory (Seqme, s.r.o., Dobříš, Czech Republic) and the sequences were manually edited and assembled (Geneious version 9.1.3, Biomatters Ltd).

Pairwise 16S rRNA gene sequence comparisons showed that strain BCCO 10_0061^T^ has pairwise similarity of 99.3, 99.2, and 99.2 % to *Lentzea californiensis* DSM 43393^T^, *Lentzea violacea* DSM 44796^T^, and *L. albidocapillata* DSM 44073^T^, respectively. Strain BCCO 10_0798^T^ had a pairwise similarity of 99.4 % to *L. flaviverrucosa* DSM 44664^T^, 99.2 % to *L. californiensis* DSM 43393^T^, and 99.2 % with *L. violacea* DSM 44796^T^, whereas strain BCCO 10_0856^T^ had a pairwise similarity of 99.2 % to *Lentzea waywayandensis* DSM 44073^T^, 99.0 % to *Lentzea rhizosphaerae* DSM 104541^T^, and 99.0 % to *Lentzea kentuckyensis* NRRL B-24416^T^. Details of mismatches and completeness are provided in Table S1, available in the online version of this article. To reconstruct a phylogenetic tree, the 16S rRNA gene sequences of the 21 available type strains (only strains representing *Lentzea* species with a validly published name according to the List of Prokaryotic Names with Standing in Nomenclature, as of 16 July 2023; Table S2) were obtained from the EzBioCloud web service [15]. The 16S rRNA gene sequences of BCCO 10_0061^T^, BCCO 10_0798^T^, BCCO 10_0856^T^, and all type strains were aligned using muscle [16]. Alignment was manually corrected, and the unrooted maximum-likelihood tree was reconstructed using RAxML-NG version 1.2.0 [17] with rapid bootstrapping on the cipres web server [18]. The final tree was visualized and edited using the iTOL editor [19].

**Fig. 1.**
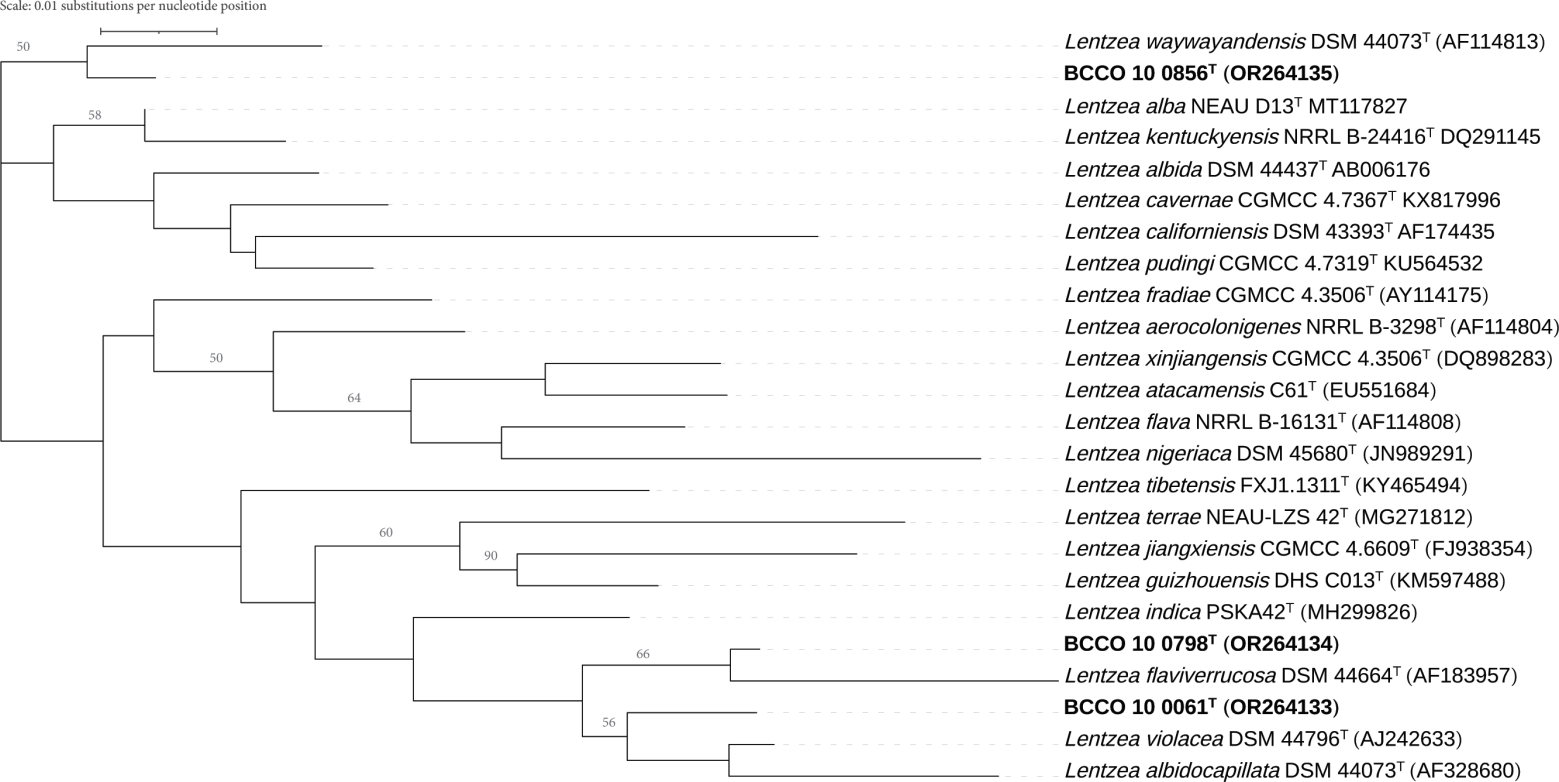
Maximum-likelihood phylogeny inferred from whole 16S rRNA gene sequences. The dataset corresponds to the 21 available type strains (only strains representing *Lentzea* species with a validly published name according to the List of Prokaryotic Names with Standing in Nomenclature as of 16 July 2023) and the BCCO strains. Tree is unrooted. Scale bar represents the length of 0.01 substitutions per nucleotide position.

Strain BCCO 10_0061^T^ clustered with *L. violacea* DSM 44796^T^ and *L. albidocapillata* DSM 44073^T^, strain BCCO 10_0798^T^ clustered with *L. flaviverrucosa* DSM 44664^T^, and strain BCCO 10_0856^T^ clustered with *L. waywayandensis* DSM 44073^T^ (Fig. 1). The topology of the maximum-likelihood tree is in concordance with previous studies [20,21]. However, a higher resolution of the phylogeny was desired as Hassler and colleagues [22] and Nakano and colleagues [23] have opened the discussion that phylogenies inferred from 16S rRNA genes alone are not sufficient to study closely related bacterial species and subspecies. Therefore, a more robust phylogenetic analysis based on multi-locus sequence typing (MLST) was performed.

## Genome features

The genomic DNA extracted from the BCCO strains was paired-end sequenced at the Genomic Research Core from the University of Illinois (Chicago, IL, USA) on an Illumina HiSeq2000. The quality of the reads was evaluated using fastQC [24] and sequences were trimmed using Trimmomatic version 0.36 [25]. Clean reads were assembled using SPAdes version 3.15.3 [26] and Velvet version 1.2.10 [27] on the KBase platform [28]. Whole-genome assemblies of the genomes of the three *Lentzea* strains were compared with those of the 21 *Lentzea* type strains obtained from the NCBI genome database (www.ncbi.nlm.nih.gov/genome/) [29], or from the EzBioCloud genome databases [15] when these were not available in NCBI. Genome completeness and contamination of all *Lentzea* genomes were assessed using CheckM version 1.1.0 [30]. Information on genome availability, nomenclature, taxonomic status, and correct reference for each of the *Lentzea* strains available in the databases is provided in Table S2. The assemblies for BCCO 10_0061^T^, BCCO 10_0798^T^, and BCCO 10_0856^T^ were initially annotated using Prokka [31] and the Kyoto Encyclopedia of Genes and Genomes Pathway database [32]. The final annotation was done using rast (Rapid Annotation using Subsystem Technology) [33]. Type strains were reannotated using rast to avoid discrepancies in annotation due to the used pipeline.

The assembled genome sequence of strain BCCO 10_0061^T^ is 10 296 987 bp long, composed of 42 scaffolds with an N50 of 882 764 bp and a DNA G+C content of 68.6 mol%. The genome encodes 9705 CDS, 69 tRNAs, and four copies of the 16S rRNA gene. The genome of the closest relative, *L. waywayandensi*s DSM 44232^T^, has a size of 10 153 412 bp, and is composed of 33 scaffolds with an N50 of 627 394 bp, and has a DNA G+C content of 68.9 mol%. The genome sequence of strain BCCO 10_0798^T^ has a size of 9 549 005 bp, is composed of 106 scaffolds with an N50 of 306 937 bp, and has a DNA G+C content of 69.2 mol%. It encodes for 9362 CDS, 68 tRNAs, and four copies of the 16S rRNA gene. The genomes of the closest relatives, *L. alba* NEAU-D13^T^ and *L. californiensis* DSM 43393^T^, are 10 211 053 and 8 998 498 bp long and have a DNA G+C contents of 67.7 and 69.3 mol%, respectively. Finally, the genome sequence of strain BCCO 10_0856^T^ has a size of 9 897 812 bp, and consists of 116 scaffolds with an N50 of 192 435 bp and a DNA G+C content of 68.5 mol%. It encodes 9408 CDS, 63 tRNAs, and three copies of the 16S rRNA gene. The closest relative, *L. kentuckyensis* NRRL B-24416^T^, has a genome size of 10 210 611 bp and a DNA G+C content of 68.7 mol%. Additional statistical data on the genome assemblies of the strains studied are shown in Table 1. For the genus *Lentzea* as a group, the average genome size is 9.5 Mb (ranging from 8.5 Mb for *Lentzea fradiae* to 10.7 Mb for *Lentzea aerocolonigenes*) with a DNA G+C content ranging from 68.3 to 70.7 mol% (according to the available genomes of 21 species). On average, the *Lentzea* reference genomes contain 8967 protein-coding genes, and 76 genes coding for rRNA. Detailed genomic information on the genome sequences of the reference strains is provided in Table S2.

**Table 1.** Characteristics of the genomes of strains BCCO 10_0061^T^, BCCO 10_0798^T^, and BCCO 10_0856^T^

Characteristic	BCCO_10 0061**^T^**	BCCO_10 0798**^T^**	BCCO_10 0856**^T^**
Contigs	42	106	116
G+C content (mol%)	68.86	69.19	68.47
Contig L50	5	11	18
Genome length (bp)	10 296 987	9 549 005	9 892 812
Contig N50	882 764	306 937	192 435
CDS	9705	9362	9408
tRNA	69	68	63
rRNA	4	4	3
Hypothetical proteins	4651	4472	4497
Proteins with functional assignments	5054	4890	4911
Proteins with EC number assignments	1503	1473	1475
Proteins with GO assignments	1294	1263	1276
Proteins with pathway assignments	1157	1121	1139

Average nucleotide identity (ANI) and digital DNA–DNA hybridization (dDDH) values were calculated using the information from whole genome assemblies with the OrthoANI [34] services of EzBioCloud [15] and the Type Strain Genome server (TYGS) [35]. ANI evaluations showed that strain BCCO 10_0061^T^ had the highest ANI value (94.0 %) with *L. waywayandensis* DSM 44232^T^. Strain BCCO 10_0798^T^ had the highest ANI value (94.3 %) with *L. albidocapillata* DSM 44073^T^. The pairwise ANI values between BCCO 10_0856^T^ and *L. alba* NEAU-D13^T^ and *L. kentuckyensis* NRRL B-24416^T^ were 89.7 and 89.6 %, respectively (Table S3). The results indicate that the overall genome relatedness between the BCCO strains and their closest relatives is below the 95 % cut-off value for grouping genomes of the same species [36]. dDDH was evaluated using the TYGS server (https://tygs.dsmz.de/) by applying the d_4_ formula. The genome sequences of BCCO_10 0061^T^ and *L. waywayandensis* DSM 44232^T^ had a dDDH value of 67.7 %. The dDDH value between BCCO 10_0798^T^ and *L. californiensis* DSM 43393^T^ was 66.2 %, and that between BCCO 10_0856^T^ and *L. waywayandensis* DSM 44232^T^ was 55.2 % (Table S4). The results of the dDDH analysis of the three BCCO strains and their closest neighbours were below the threshold of 70 % established for delineating prokaryotic species [37], suggesting that each of the strains represents a new species of the genus *Lentzea*.

## Core genome MLST

The relationships based on core genome MLST (cgMLST) of BCCO 10_0061^T^, BCCO 10_0798^T^, and BCCO 10_0856^T^ were evaluated against the same 21 strains used for 16S rRNA typing. We performed core genome multi-locus sequence typing based on 100 single-copy genes from the core genome of our *Lentzea* dataset. cgMLST aims to take advantage of the discriminatory power from extensive genetic data derived from whole genome sequencing hence providing maximum-resolution phylogenomic analyses (up to strain level) and has been used in clinical microbiology to type clonal outbreaks [38,44]. The core genome was identified by Up-to-date Bacterial Core Genes 2 pipeline [45] and re-annotated with InterPro [46]. Once annotated, the core genes were aligned and the best alignment scores and mean variability per position were evaluated for all gene families. The best 100 scored families were used for the reconstruction of the tree (Table S5). The tree was reconstructed using RAxML [17] with rapid bootstrapping. The final tree was visualized and edited using the iTOL editor [19] The resulting clusters position strain BCCO 10_0061^T^ as being closely related to *L. waywayandensis* DSM 44232^T^; strain BCCO 10_0798^T^ as closely related to *L. albidocapillata* DSM 44073^T^, *L. flaviverrucosa* DSM 44664^T^, *L. californiensis* DSM 43393^T^ and *L. violacea* DSM 44796^T^; and strain BCCO 10_0856^T^ appeared in a distinct branch closely related to *L. kentuckyensis* NRRL B-24416^T^ and *L. alba* NEAU-D13^T^ (Fig. S1). These results are consistent with the tree previously presented by [5,29], who also constructed their phylogenetic tree using the core protein-coding genes of the available genomes.

Based on 16S rRNA gene sequence similarities and the results of the MLST, *L. waywayandensis* DSM 44232^T^ was selected as the reference strain for the polyphasic taxonomy of BCCO 10_0061^T^; *L. alba* NEAU-D13^T^ and *L. kentuckyensis* NRRL B-24416^T^ served as reference strains for BCCO 10_0856^T^; and *L. californiensis* DSM 43393^T^ for strain BCCO 10_0798^T^, respectively.

## Inference of metabolic potential

The rast annotation file from the draft genome assembly was used as the basis for biosynthetic gene cluster (BGC) prediction using AntiSMASH [47]. The strains were screened for secondary metabolite BGCs using the antiSMASH version 7.0 server (accessed July 2023). The analysis resulted in the identification of 17 BGCs in strain BCCO 10_0856^T^, 22 BGCs in BCCO 10_0061^T^, and 20 BGCs in BCCO 10_0798^T^. We present and discuss here the results of BGCs with homologies between 50 and 100 % to known BGCs.

Terpenes were the most abundant type of BGCs with two clusters for geosmin (100 % homology) [48,49], being present in all strains. Geosmin is a volatile, earthy-smelling, interspecies signalling molecule [48,49] that seems to have a very important role in spore dispersion of *Streptomyces* species [50]. All of our strains also carry a BGC for the production of coelichelin (72 % homology) a siderophore with high affinity for iron [51]; a BGC for RiPP:Lanthipeptide class III (75 % homology), which probably codes for the synthesis of various Ery-9 molecules, as well as a BGC for ε-poly-l-lysine (100 % homology), a natural antimicrobial cationic peptide [52]

We found only in BCCO 10_0061^T^ and BCCO 10_0798^ T^ a BGC for isorenieratene (85 % homology), which is a carotenoid and photooxidation quencher [53]; and a BGC for 2-methylisoborneol (50 % homology), which is a volatile, interspecies signalling molecule similar to and usually found with geosmin [50].

Polyketide BGCs were represented by a nystatin A1 BGC (present in BCCO 10_0061^T^ with 78 % identity and in BCCO 10_0856^T^ with 72 % homology) and an *iso*-migrastatin BGC in BCCO 10_0061^T^. *iso*-Migrastatin is one of the most potent inhibitors of human tumour cell migration and thus represents an anticancerours compound [54]. In addition, the BGC for staurosporine, an alkaloid that acts as a nonselective inhibitor of protein kinases with apoptotic activity, was identified only in the genome of BCCO 10_0798^T^ (Table S6). The production of yellow to reddish brown diffusible pigment in the media by this strain and the colour of the mycelium may indicate its biosynthesis according to [55,55] (Fig. S2).

## Physiology and chemotaxonomy

The pigmentation of the strains and production of aerial mycelium were determined after 14 days of incubation at 28 °C on yeast extract–malt extract agar ISP 2 (DSMZ 987), oatmeal agar ISP 3 (DSMZ 609), and inorganic salts–starch agar ISP 4 (DSMZ 547), as described by [12,12], and on GYM *Streptomyces* medium (DSMZ 65 [13]). Melanoid pigment production was determined on peptone–yeast extract agar ISP 6 (DSM 1269) and tyrosine agar ISP 7 (DSM 1619), prepared as described by [12,12]. The optimal growth conditions regarding temperature (4, 10, 17, 20, 25, 28, 35, 37, 42, and 45 °C) and pH growth range (pH 4–13 with increments of 1 pH unit) were tested on GYM *Streptomyces* medium (DSMZ 65 [13]) in duplicate. The pH values were adjusted using NaOH or HCl and all pH values of the prepared media were checked after autoclaving and adjusted if necessary. Growth occurred in the temperature range between 10 and 37 °C, which corresponded to the reported temperature range of their closest relatives (Table 2). However, the optimal growth temperature of BCCO 10_0798^T^ and BCCO 10_0856^T^ was 35 °C, in contrast to BCCO 10_0061^T^ and its closest relatives, for which the optimal temperature was 28 °C or lower [20,56, 57]). All strains described in this study were highly tolerant to alkaline conditions, with minor growth occurring even at pH 13.

**Table 2.** Differential characteristics of strains BCCO 10_0061^T^, BCCO 10_0798^T^, BCCO 10_0856^T^ and *Lentzea* type strains Strains: 1, *L. alba* NEAU-D13^T^; 2, *L. californiensis* DSM 43393^T^; 3, *L. kentuckyensis* NRRL B-24416^T^; 4, *L. waywayandensi*s DSM 44232^T^; 5, BCCO 10_0061^T^; 6, BCCO 10_0798^T^; 7, BCCO 10_0856^T^. Data were obtained from Sun *et al*.[57] for *L. alba* and *L. kentuckyensis*; and from Labeda *et al*. [20] for *L. californiensis* and *L. waywayandensis.*

Characteristic	1	2	3	4	5	6	7
Isolation source	Soil, China	Soil, California	Equine placenta	Soil, New Jersey	Bare soil, Czech Republic	Bare soil, Czech Republic	Bare soil, Czech Republic
DNA G+C content (mol%)	68.7	69.3	68.8	68.9	68.8	69.2	68.5
Temperature range (optimum) for growth (°C)	10–40 (28)	10–37 (28)	10–37 (23.5)	15–37 (28)	10–37 (28)	10–37 (35)	10–37 (35)
pH range (optimum) for growth	5–9	n.d.	n.d.	5–10	5–13	5–13	5–13
Morphology on ISP 3 (oat meal agar)	Soluble pigments: Light yellowish brown, greyish greenish yellow, strong yellowish brown	Soluble pigments: none	Soluble pigments: none	Soluble pigments: none	Soluble pigments: none	Soluble pigments: dark reddish brown	Soluble pigments: none
Menaquinones	MK-9(H_4_)	MK-9(H_4_)	MK-9(H_4_)	MK-9(H_4_)	MK-9(H_4_)	MK-9(H_4_)	MK-9(H_4_)
Diagnostic diamino acid	*meso*-Diaminopimelic acid	*meso*-Diaminopimelic acid	*meso*-Diaminopimelic acid	*meso*-Diaminopimelic acid	*meso*-Diaminopimelic acid	*meso*-Diaminopimelic acid	*meso*-Diaminopimelic acid
Whole-cell sugars	Ribose, mannose	Rhamnose, galactose	Galactose, ribose	Rhamnose, galactose	Rhamnose, ribose, mannose, glucose, and galactose	Rhamnose, ribose, mannose, glucose, and galactose	Rhamnose, ribose, mannose, glucose, and galactose
Phospholipid profile	DPG, PE, OH-PE, PI	PE, DPG, PG, PI	DPG, PE, OH-PE, PI, PG (traces), PIM (traces), two unknown glycolipids	PE major, phospholipid pattern PII	Methyl-PE, PE, OH-PE DPG, PG, PI and other unknown GPL, PL and L	Methyl-PE, PE, OH-PE, DPG, PG, PI and other unknown GPL, PL and L	Methyl-PE,PE, OH-PE,DPG, PG, PI and other unknownGPL, PL and L
Major fatty acids (>10%)	iso-C_15 : 0_, anteiso-C_15 : 0_, iso-C_16 : 0_, C_16 : 0_, anteiso-C_17 : 0_	iso-C_14 : 0_, anteiso-C_15 : 0_, iso-C_16 : 0_, C_16 : 0_	iso-C_15 : 0_, anteiso-C_15 : 0_, iso-C_16 : 0_, C_16 : 0_, anteiso-C_17:0_	iso-C_14 : 0_, iso-C_16 : 1_, iso-C_16 : 0_	iso-C_15 : 0_, anteiso-C_15 : 0_, iso-C_16 : 0_, C_16 : 0_	iso-C_15 : 0_, anteiso-C_15 : 0_, iso-C_16 : 0_, C_16:0,_ C_16 : 1_ ω7*c*	iso-C_15 : 0_, anteiso-C_15 : 0_, iso-C_16 : 0_, C_16 : 0_
Carbon source utilization:							
Cellulose	nd	+	+	nd	−	−	−
d-Fructose	−	+	+	+	+/−	+/−	+/−
d-Glucose	−	+	+	+	+	+	+
d-Mannose	−	+	+	+	+	+	+
Raffinose	+	+	+/−	+/−	−	+	−
d-Xylose	−	+	+	+	+	+	+
*i*-Inositol	nd	nd	nd	nd	+/−	+/−	+/−
l-Arabinose	−	+	+	+	+	+	+
l-Rhamnose	−	+	+	+	−	+	+
Sucrose	nd	+	+/−	+	+	+	+
Enzyme activity:							
Lipase (C14)	nd	nd	nd	nd	+/−	+	−
β-Glucuronidase	nd	nd	nd	nd	−	+/−	+
α-Fucosidase	nd	nd	nd	nd	−	−	+/−

– negative +/– weakly positive or inconclusive + Positive DPG diphosphatidylglycerol GPL glycophospholipids L lipids Methyl-PE methyl-phosphatidylethanolamin
nd
not determined or no data OH-PE hydroxy-phosphatidylethanolamine PE phosphatidylethanolamine PG phosphatidylglycerol PI phosphatidylinositol PIM phosphatidylinositol mannosides PL phospholipids

**Fig. 2.**
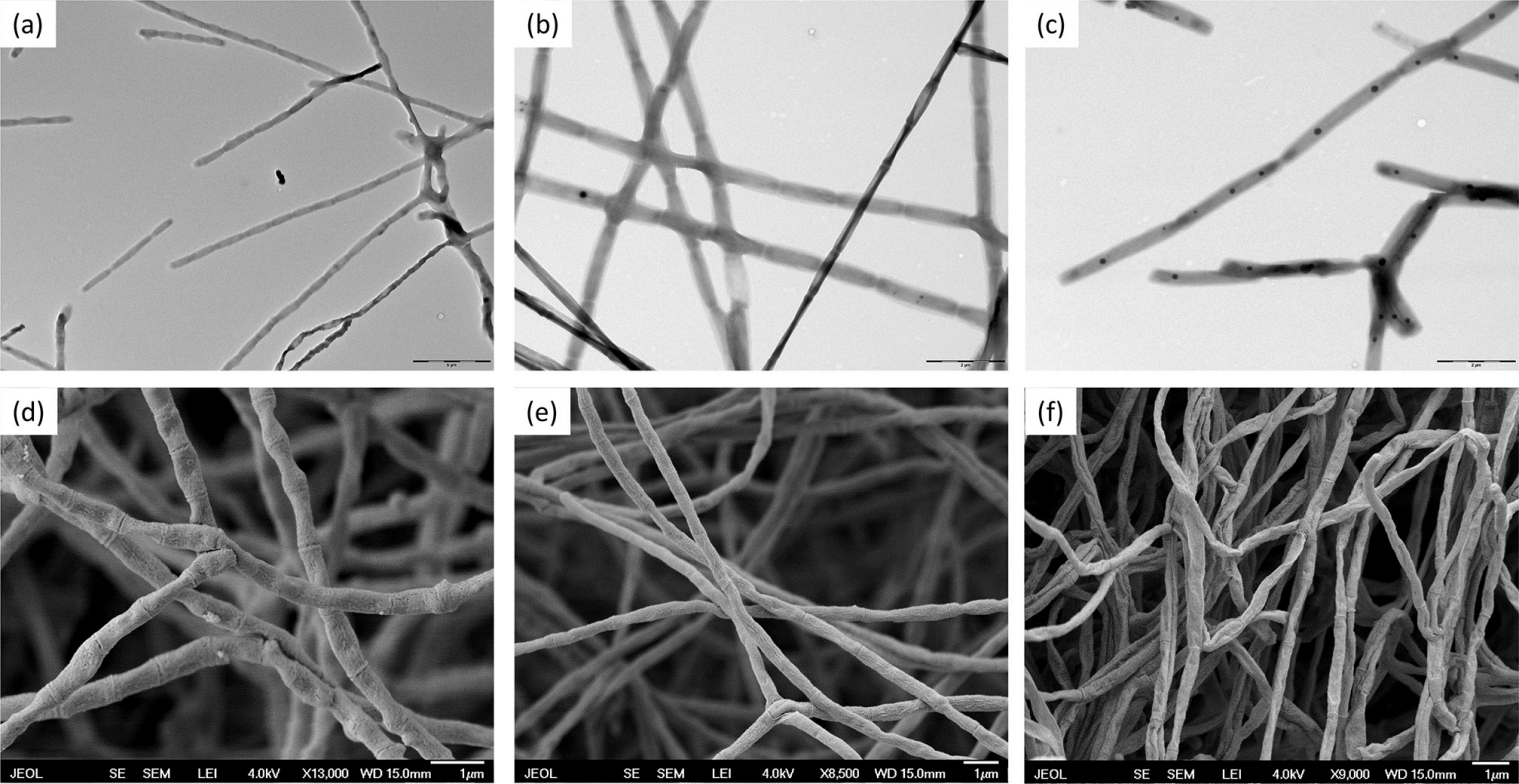
Electron microphotography (EM) of the spore mass and aerial hyphae of the strains BCCO 10_0061^T^ (a, d), BCCO 10_0798^T^ (b, e) and BCCO 10_0856^T^.(c, f) Transmission EM (a, b, c) and scanning EM (d, e, f).

The micromorphology of the organism was determined by using a culture grown at 28 °C for 14 days on ISP 2. The morphology of the sporophores produced by the studied strains was analysed with the scanning electron microscope jeol 7401-FE at the accelerating voltage of 4 kV. The sample (aerial mycelium) was fixed and dehydrated in vapours of OsO4, then frozen and, after evaporation, coated with gold using a Sputter Coater (Baltec-SCD 050). The spore shape, size and surface were classified by the examination of carbon replicas of spore mass with the transmission electron microscope (jeol 1010) at the acceleration voltage of 80 kV with a Mega View III camera (SIS). Strains BCCO 10_0061^T^, BCCO 10_0798^T^, and BCCO 10_0856^T^ were able to grow and sporulate on all typical *Streptomyces* culture media tested. Morphological observation revealed that strains BCCO 10_0061^T^, BCCO 10_0798**^T^**, and BCCO 10_0856^T^ exhibited typical characteristics of the genus *Lentzea*. The aerial mycelium was well developed and fragmented into rod-shaped, thin, and long spores (Fig. 2). The length and width of the spores ranged from 1.5 to 1.8 µm and from 0.4 to 0.5 µm, respectively (median values are indicated). The spore surface was smooth in all three cases. The strains developed straight to flexuous sporophores [58].

Strain BCCO 10_0061^T^ produced vegetative mycelium of cream to light brown colour (Coo5s) and branched aerial hyphae on all culture media tested. The colour of the aerial mycelia was pale yellow (Y2ba). No soluble pigment was produced. The morphological appearance of BCCO 10_0798^T^ is unique among members of the genus *Lentzea*, and changes depending on the culture medium. On ISP 2 medium and GYM it produced cream to brown (Coo5s) vegetative mycelium, whereas on ISP 3 and ISP 4 media it produced dark reddish brown (O5r) or orange-brown (Oc4b,4s) vegetative mycelia, respectively. The dark brown or orange-ochre-pink coloured soluble pigment was produced only on ISP 3 and ISP 4, respectively. The production of the dark soluble pigment distinguished this strain from its closest relatives, *L. alba* NEAU-D13^T^, which produces a light pigment, and *L. californiensis* DSM 43393^T^, which produces no soluble pigment at all (Fig. S2). The aerial mycelium was pale yellow (Y2ba). Strain BCCO 10_0856^T^ produced vegetative mycelium of pale yellow (Coo5a) to orange-brown (Coo4a,4b) colour on media ISP 2, ISP 3, ISP 4 and GYM. The aerial mycelium was pale yellow (Y2ba). None of the strains produced melanin pigment on peptone–yeast extract iron agar (ISP 6) or tyrosine agar (ISP 7).

Biomass for the chemotaxonomic analysis was prepared as follows: A preculture grown in 20 ml ISP 2 medium for 48 h (150 r.p.m., 28 °C) served as inoculum for 100 ml ISP 2 main culture, which was grown for 48 h at 150 r.p.m. and 28 °C. Biomass was harvested by centrifugation (10 min, 3 000 r.p.m.), washed twice with sterile PBS and freeze-dried (L10-55 PRO, Gregor Instruments). Freeze-dried biomass was examined for the whole-cell sugar composition [59], polar lipid pattern [60], and diaminopimelic acid isomer form of the peptidoglycan [61], and detected by TLC. The cellular fatty acid analysis was performed by a GC-MS run on an Agilent GC-MS 7000D instrument as described by Vieira *et al*. [62]. An HPLC coupled to a diode array detector and high-resolution mass spectrometer was used for isoprenoid quinones analysis [63]. The chemotaxonomic characteristics of strains BCCO 10_0061^T^, BCCO 10_0798^T^, and BCCO 10_0856^T^ correspond to those of other members of the genus *Lentzea* [56].

All strains described in this study contained *meso*-diaminopimelic acid in their peptidoglycan as well as galactose, mannose, and ribose in whole-cell hydrolysates; diphosphatidylglycerol (DPG), phosphatidylinositol (PI), phosphatidylglycerol (PG), and phosphatidylethanolamine (PE) as phospholipid constituents corresponding to the phospholipid type II according to Lechevalier *et al*.[64], and MK-9 (H_4_) as the predominant menaquinone (87.9 % in BCCO 10_0061^T^, 90.6 % in BCCO 10_0798^T^, and 82.8 % in BCCO 10_0856^T^). MK-9 (H_2_) and MK-9 (H_6_) were also found; however, they were in the minority (4.1 –11.3 % and 3.6 –5.9 %, respectively).

When the BCCO strains were compared with their closest relatives, differences in chemotaxonomic markers were observed. The whole-cell hydrolysates of the BCCO strains contained galactose, mannose, ribose, rhamnose, and glucose, whereas *L. waywayandensis* DSM 44232^T^ contained rhamnose and galactose [20], *L. alba* NEAU-D13^T^ contained ribose and mannose [57], *L. kentuckyensis* NRRL B-24416^T^ contained rhamnose and galactose [57], and *L. californiensis* DSM 43393^T^ contained galactose and ribose [20]. In addition to DPG, PI, PG, and PE in the polar lipid pattern of the three BCCO strains, they contained methyl-phosphatidylethanolamine (Methyl-PE) and unidentified phospholipids, glycophospholipids, and lipids (Fig. S3), distinguishing them from their closest relatives. Notably, *L. waywayandensis* DSM 44232^T^ contained only PE as the major diagnostic phospholipid [20], whereas BCCO 10_0061^T^ had a more diverse phospholipid profile. BCCO 10_0798^T^ differed from *L. alba* by the presence of Methyl-PE and from *L. californiensis* DSM 43393^T^ by the presence of Methyl-PE and PG in the cell wall [20]. BCCO 10_0856^T^ differed from *L. kentuckyensis* NRRL B-24416^T^ by the presence of Methyl-PE [57] and a greater amount of PG, while phosphatidylinositol mannosides (PIM) were absent in the whole-cell hydrolysate. The presence of hydroxy-phosphatidylethanolamine (OH-PE) in BCCO strains was uncertain.

The predominant fatty acids (>10%) in all BCCO strains were iso-C_15 : 0_, anteiso-C_15 : 0_, iso-C_16 : 0_, and C_16 : 0_, with additional C_16 : 1_ ω7*c* in strain BCCO 10_0798^T^. The complete fatty acid profiles of the BCCO strains are in Table S7. The composition of major fatty acids of the closest relatives differed from those of the BCCO strains: BCCO 10_0061^T^ contained iso-C_15 : 0_, anteiso-C_15 : 0_, iso*-*C_16 : 0_, and C_16 : 0_, while *L. waywayandensi*s DSM 44232^T^ contained only *i*so-C_14 : 0_, iso-C_16 : 1_, and iso-C_16 : 0_ as major fatty acids [20]. BCCO 10_0798^T^ contained iso-C_15 : 0_, C_15 : 0_, iso-C_16 : 0_, anteiso-C_16 : 0_, and C_16 : 1_ ω7*c*, while *L. californiensis* DSM 43393^T^ contained iso-C_14 : 0_, anteiso-C_15 : 0_, iso-C_16 : 0_, and C_16 : 0_ as major fatty acids [20]. Finally, BCCO 10_0856^T^ contained iso-C_15 : 0_ iso-C_16 : 0_, anteiso-C_15 : 0_, and C_16 : 0_, while *L. kentuckyensis* NRRL B-24416^T^ and *L. alba* NEAU-D13^T^ also contained anteiso-C_17 : 0_ as a major fatty acid [57].

The enzymatic profiles of the strains were determined by API ZYM tests (bioMérieux) following the instructions of the manufacturer. All three BCCO strains produced alkaline phosphatase, esterase, esterase lipase, cystine, valine, and leucine arylamidase, trypsin, α-chymotrypsin, acid phosphatase, naphthol-AS-BI-phosphohydrolase, α- and β-galactosidase, α- and β-glucosidase, *N*-acetyl-β-glucosaminidase, and α-naphthyl-*N*-acetyl-β-d-glucosaminide. Enzyme activity differed between strains for lipase, β-glucuronidase, and α-fucosidase (Table 8).

The utilization of different carbon sources (glucose, arabinose, mannose, inositol, rhamnose, fructose, raffinose, cellulose, sucrose, and xylose) was evaluated on ISP 2 agar after 3, 7, 10, and 13 days at 28 °C, in duplicate [12]. All three BCCO strains were able to utilize d-glucose (positive control), d-mannose, d-xylose, l-arabinose, and sucrose, and weakly utilized d-fructose and *i*-inositol. Results for cellulose degradation were inconclusive as no growth was detected. Differences in the utilization of l-rhamnose and raffinose were found between the three BCCO strains: l-rhamnose was utilized by BCCO 10 0798^T^ and BCCO 10_0856^T^, but not by BCCO 10_0061^T^; raffinose was utilized by BCCO 10 0798^T^, but not by BCCO 10 0061^T^ or BCCO 10_0856^T^. In comparison with the reference strains, BCCO 10_0061^T^ cannot utilize cellulose, raffinose and l-rhamnose, which differentiated it from *L. waywayandensi*s DSM 44232^T^. BCCO 10_0798^T^ could utilize similar sugars to *L. californiensis* DSM 43393^T^ with the exception of cellulose, but its utilization pattern was very different from that of * L. alba* NEAU-D13^T^, which could only utilize raffinose of all compounds tested in our study. BCCO 10_0856^T^ cannot utilize raffinose, which distinguishes BCCO 10_0856^T^ from *L. kentuckyensis* NRRL B-24416^T^.

## Description of *Lentzea sokolovensis* sp. nov.

*Lentzea sokolovensis* (so.ko.lo.ven.sis, N.L. fem. adj. *sokolovensis*, of Sokolov, named after the place of origin of the type strain, the town of Sokolov, Czech Republic).

Gram-positive. Substrate mycelium is cream to brown on ISP 2, ISP 3 and ISP 4. Dense, white to yellowish-white aerial mycelium is produced on all culture media tested. Melanin pigment is not produced on either ISP 6 or 7. No soluble pigments are produced. Sporophores are straight to flexuous, spores are rod-shaped, 1.5 µm long and 0.5 µm wide (median values). d-Glucose, d-mannose, d-xylose, l-arabinose and sucrose are utilized, d-fructose and i-inositol are weakly utilized, and l-raffinose, l-rhamnose, and cellulose are not utilized.

Alkaline phosphatase, acid phosphatase, esterase, esterase lipase, cystine, valine and leucine arylamidase, trypsin, α-chymotrypsin, naphthol-AS-BI-phosphohydrolase, α- and β-galactosidase, α- and β-glucosidase, *N*-acetyl-β-glucosaminidase, and α-naphthyl-*N*-acetyl-β-d-glucosaminide are produced, weak production of lipase, and no activity of β-glucuronidase and α-fucosidase is observed.

Grows at 10–37 °C, with optimal growth at 28 °C. Grows at pH 5–12, with weak growth at pH 13. The cell wall is of the III type (*meso*-DAP). The whole-cell sugar pattern consists of rhamnose, ribose, mannose, glucose, and galactose. Possesses a type II phospholipid pattern (PE, OH-PE, methyl-PE, DPG, PG, PI and other unknown GPL, phospholipids and lipids). The major menaquinone is MK-9 (H_4_). Mycolic acids are absent. The predominant fatty acids (>10 %) are iso-C_15 : 0_, anteiso-C_15 : 0_, iso-C_16 : 0_, and C_16 : 0_. The G+C content of the DNA is 68.8 mol%, and the genome size is 10.3 Mb.

The type strain, BCCO 10_0061^T^ (=DSM 116175^T^), was isolated from a soil sample collected in Sokolov Coal Basin, Czech Republic. The GenBank accession number for the 16S rRNA gene sequence of strain BCCO 10_0061^T^ is OR264133. The whole genome shotgun project has been deposited in GenBank under accession number SRR25230625.

## Description of *Lentzea kristufekii* sp. nov.

*Lentzea kristufekii* (Kris.tu.fe’kii, N.L. fem. adj. *kristufekii* of Kristůfek, named after the Czech microbiologist Václav Krištůfek, who contributed substantially to the biology and ecology of actinomycetes during his work at the Institute of Soil Biology, Biology Centre CAS).

Gram-positive. The substrate mycelium is cream to brown on ISP 2, but on ISP 3 and ISP 4 it develops a reddish-brown or an orange-brown coloration, respectively. Dense, pale yellow aerial mycelium is produced on all culture media tested, except on ISP 4, where the aerial spore mass is light greyish reddish brown. Melanin pigment is not produced on either ISP 6 or ISP 7. An orange-ochre-pink soluble pigment is produced on medium ISP 4. Sporophores are straight to flexuous, and spores are rod-shaped, 1.7 µm long and 0.5 µm wide (median values). d-Glucose, d-mannose, d-xylose, l-arabinose, raffinose, l-rhamnose, and sucrose are utilized, d-fructose and I-inositol are weakly utilized, and cellulose is not utilized.

Alkaline phosphatase, acid phosphatase, esterase, esterase lipase, cystine, valine and leucine arylamidase, trypsin, α-chymotrypsin, naphthol-AS-BI-phosphohydrolase, α- and β-galactosidase, α- and β-glucosidase, *N*-acetyl-β-glucosaminidase, α-naphthyl-*N*-acetyl-β-d-glucosaminide and lipase are produced, but only weak production of β-glucuronidase and no activity of α-fucosidase is observed.

Grows at 10–37 °C, with optimum growth at 35 °C. Grows at pH 5–13. The cell wall is of the III type (*meso*-DAP). Whole-cell sugar pattern consists of rhamnose, ribose, mannose, glucose, and galactose. Possesses the type II phospholipid pattern (PE, OH-PE, methyl-PE, DPG, PG, PI and other unknown GPL, phospholipids and lipids). The menaquinone is MK-9 (H_4_). Mycolic acids are absent. The predominant fatty acids (>10 %) are iso-C_15 : 0_, anteiso-C_15 : 0_, iso-C_16 : 0_, C_16 : 0_, and C _16 : 1_ ω7*c*. The G+C content of the DNA is 69.2 mol%, and the genome size is 9.5 Mb.

The type strain, BCCO 10_0798^T^ (=DSM 116176^T^), was isolated from a soil sample collected in Sokolov Coal Basin, Czech Republic. The GenBank accession number for the 16S rRNA gene sequence of strain BCCO 10_0798^T^ is OR264134. The whole genome shotgun project has been deposited in GenBank under accession number SRR25230624.

## Description of *Lentzea miocenica* sp. nov.

*Lentzea miocenica* (mio.ce’ni.ca, N.L. fem. adj. *miocenica*, pertainingto the Miocene clay, named after the source of origin of the type strain, Miocene lacustrine clay sediment, Sokolov Brown Coal Basin, Czech Republic).

Gram-positive. Substrate mycelium is pale yellow to orange brown on ISP 2, ISP 3 and ISP 4, but ochre on ISP 6. Dense, white to yellowish white aerial mycelium is formed on all media tested. Melanin pigment is not produced on ISP 6 or 7. No soluble pigments are produced. Sporophores are straight to flexuous, spores are rod-shaped, 1.8 µm long and 0.4 µm wide (median values). d-Glucose, d-mannose, d-xylose, l-arabinose and sucrose are utilized, d-fructose and i-inositol are weakly utilized, and raffinose, l-rhamnose, and cellulose are not utilized.

Alkaline phosphatase, acid phosphatase, esterase, esterase lipase, cystine, valine and leucine arylamidase, trypsin, α-chymotrypsin, naphthol-AS-BI-phosphohydrolase, α- and β-galactosidase, α- and β-glucosidase, *N*-acetyl-β-glucosaminidase, α-naphthyl-*N*-acetyl-β-d-glucosaminide and β-glucuronidase are produced, but only weak production of α-fucosidase, and no activity of lipase is observed.

Grows at 10–37 °C, with optimal growth at 35 °C. Grows at pH 5–12, with weak growth at pH 13. The cell wall is of type III (*meso*-DAP). The whole-cell sugar pattern consists of rhamnose, ribose, mannose, glucose, and galactose. Possesses a type II phospholipid pattern PE, OH-PE, methyl-PE, DPG, PG, PI and other unknown GPL, phospholipids and lipids). The major menaquinone is MK-9 (H_4_). Mycolic acids are absent. The predominant fatty acids (>10 %) are iso-C_15 : 0_, anteiso-C_15 : 0_, iso-C_16 : 0_, and C_16 : 0_. The G+C content of the DNA is 68.5 mol%, and the genome size is 9.9 Mb.

The type strain, BCCO 10_0856^T^ (=DSM 116177^T^), was isolated from a soil sample collected in Sokolov Coal Basin, Czech Republic. The GenBank accession number for the 16S rRNA gene sequence of strain BCCO 10_0856^T^ is OR264135. The whole genome shotgun project has been deposited in GenBank under accession number SRR25230626.

## supplementary material

10.1099/ijsem.0.006335 Uncited Supplementary Material 1.
