# Supplementary material for: Lentzea sokolovensis sp. nov., Lentzea kristufekii sp. nov. and Lentzea miocenica sp. nov., rare actinobacteria from Miocene lacustrine sediment of the Sokolov Coal Basin, Czech Republic
Source: Int J Syst Evol Microbiol. 2024 Apr 17;74(4):006335. doi: 10.1099/ijsem.0.006335 (PMC11092160; doi:10.1099/ijsem.0.006335)
Supplement: Uncited Supplementary Material 1. [file ijsem-74-06335-s001.pdf]

SUPPLEMENTARY MATERIAL

***Lentzea sokolovensis*, sp. nov., *Lentzea kristufekii*, sp. nov., and *Lentzea miocenica*, sp. nov., rare actinobacteria from Sokolov Coal Basin, Miocene lacustrine sediment, Czech Republic**

Ana Catalina Lara [1,2]; Lucie Kotrbová [1,3]; Moritz Keller[1,3]; Imen Nouioui [4]*, Meina Neumann-Schaal[4], Yvonne Mast[4], Alica Chroňáková[1]*

1-Biology Centre Czech Academy of Sciences, Institute of Soil Biology and Biogeochemistry, Na Sádkách 7, 37005 České Budějovice, Czech Republic

2-University of Chemistry, and Technology, Prague, Faculty of Food and Biochemical Technology, Department of Biochemistry and Microbiology, Technická 5, 16628 Prague, Czech Republic

3-University of South Bohemia, Faculty of Science, Branišovská 31, 37005 České Budějovice, Czech Republic

4- Leibniz Institute DSMZ - German Collection of Microorganisms and Cell Cultures, Inhoffenstraße 7B, 38124 Braunschweig, Germany

1*Corresponding author: RNDr. Alica Chroňáková, PhD., alica.chronakova@upb.cas.cz

4*Corresponding author: Dr. Imen Nouioui, ino20@dsmz.de

This PDF file includes: 3 Figures and 8 Tables.

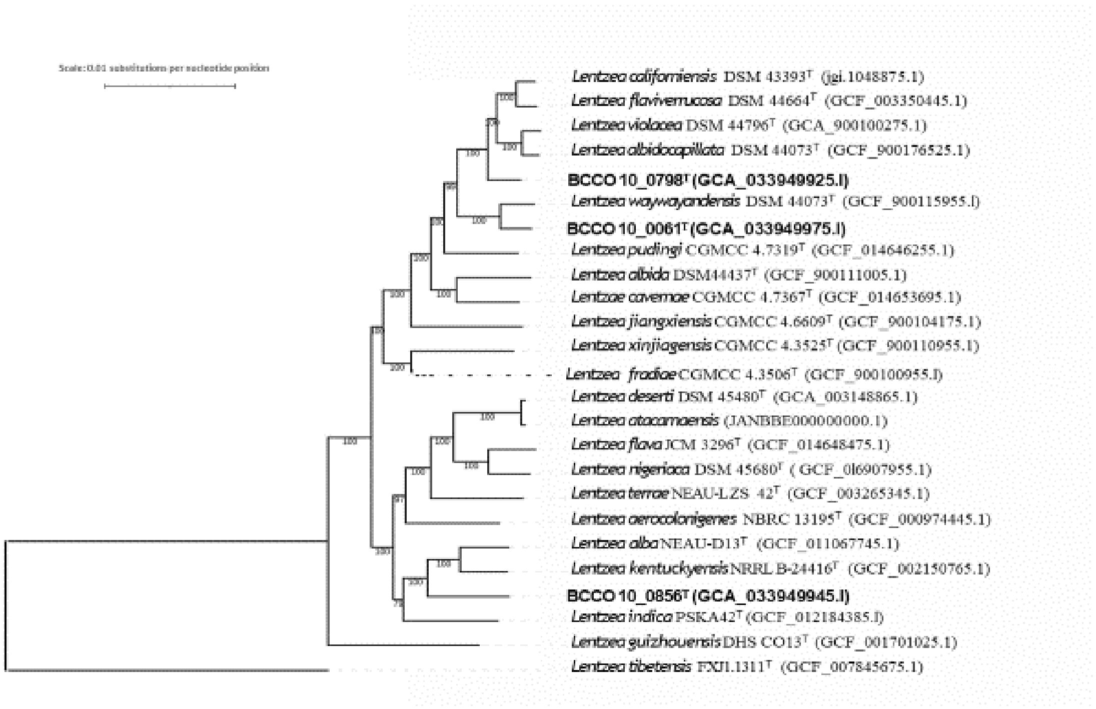

Supplementary Figure 1: Maximum likelihood tree based on 100 concatenated single copy genes showing the relationships between strains BCCO 10_0061^T, BCCO 10_0798^T, BCCO 10_0856^T and the 21 phylogenetically closely related species with validly-published names in the genus *Lentzea*. Only bootstrap values above 50% are indicated. Scale bar represents lenght of 0.01 substitutions per nucleotide position.

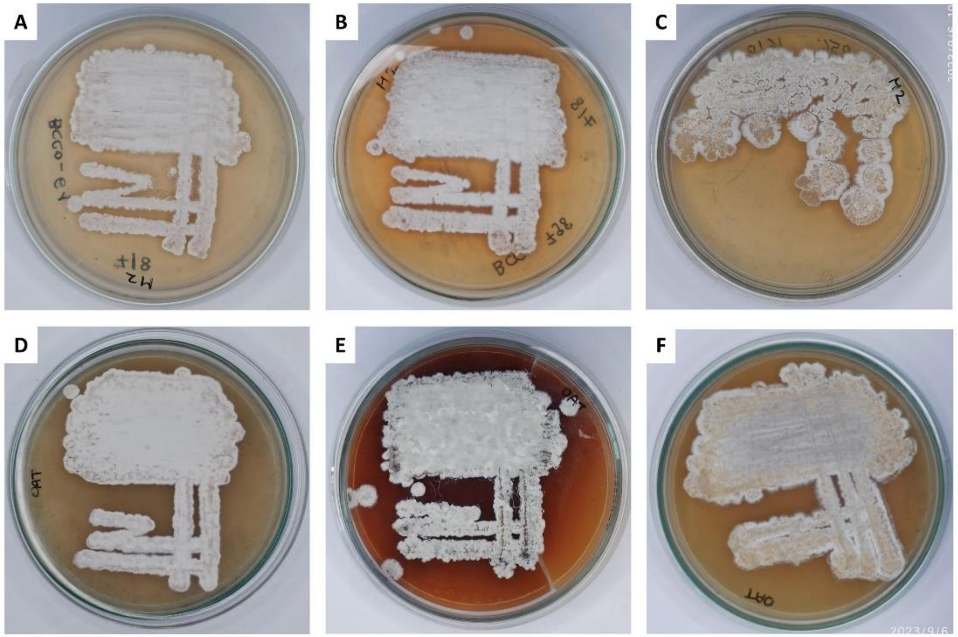

ISP2 medium

ISP3 medium

Supplementary Figure 2: The production of yellow to reddish brown diffusible pigment in the media (panel E) by strain BCCO 10_0798^{T} and the color of the mycelium may indicate biosynthesis of of staurosporine.

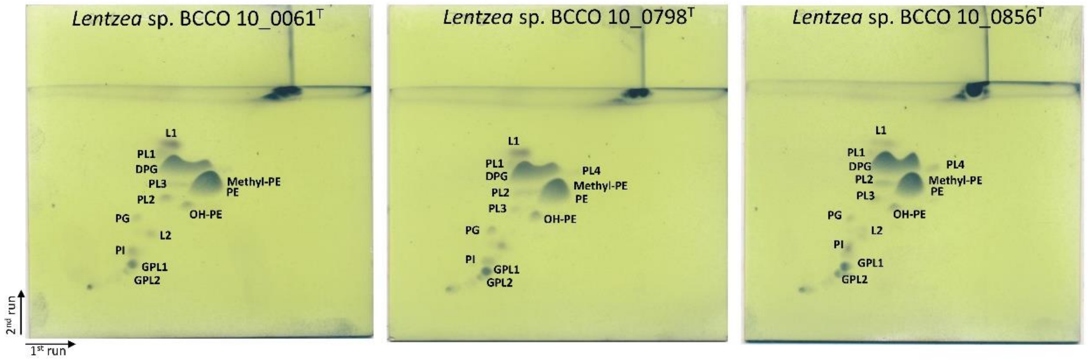

Supplementary Figure 3. Two-dimensional thin layer chromatography of polar lipids of *Lentzea* sp. isolates BCCO 10_0061^T, BCCO 10_0798^T and BCCO 10_0856^T. 30 µl of each extract was applied on silica HPTLC plates (Merck .05631.0001) and developed in solvent 1 (chlorophorm-methanol-water; 65:254) in the first direction followed by solvent 2 (chlorophormacetic acid-methanol-water; 80:15:12:4) in the second direction. The results were visualised by phosphomolybdic acid spray (Sigma 319279). Abbreviation: DPG - diphosphatidylglycerol, PE - phosphatidylethanolamine, OH-PE - hydroxy-phosphatidylethanolamine, Methyl-PE - methyl-phosphatidylethanolamin, PG - phosphatidylglycerol, Pl - phosphatidylinositol, GLP - glycophosphospholipid, PL - phospholipide, L - lipide.

Supplementary Table 1. Comparisons of 16S rDNA sequences between BCCO strains and the three more closely related type strains.

Strain	The closest relatives	Pairwise Similarity (%)	Mismatch/ Total nt	Completeness (%)
**BCCO_10 0061^T**	*Lentzea californiensis* DSM 43393 ^{T}	99.29	10/1409	100
	Lentzea violacea DSM 44796 ^{T}	99.22	11/1407	100
	Lentzea albidocapillata DSM 44073 ^{T}	99.15	12/1409	100
**BCCO_10 0798^T**	*Lentzea flaviverrucosa* DSM 44664 ^{T}	99.35	9/1395	98
	Lentzea californiensis DSM 43393 ^{T}	99.15	12/1411	100
	Lentzea violacea DSM 44796 ^{T}	99.15	12/1409	100
**BCCO_10 0856^T**	*Lentzea waywayandensis* DSM 44232 ^{T}	99.15	12/1409	100
	Lechevalieria rhizosphaerae DSM 104541 ^{T}	99.01	14/1410	100
	Lentzea kentuckyensis DSM 44909 ^{T}	99.01	14/1410	100

Supplementary Table 2. The information on the strains used for phylogenetic analyses. Data on the species name, strain number, nomenclatural status, taxonomy status, origin of the strains. In addition, it includes data on the genome availability, source database (NCBI or EzTaxon), accession numbers and basic characteristics of genomes. References (to description of the strain and to publication of the genome when the the strain was described in a different publication to the genome publications) are listed below the table. Abbreviations: ICNP - The International Code of Nomenclature of Prokaryotes, TYGS - Type (Strain) Genome Server (https://tygs.dsmz.de/), Y - yes, genome was available, N- no, genome was not available.

Species name	Strain No.	Reference for species description and reclassification	Nomenclatural status	Taxonomic status	Origin	Genome available June 2022	Reference genome for TYGS
Lentzea aerocolonigenes	NBRC 13195	(Labeda 1986) Nouioui et al. 2018	validly published under the ICNP	correct name	soil	Y	Y
Lentzea alba	NEAU-D13	Sun et al. 2021	validly published under the ICNP	correct name	soil	Y	Y
Lentzea albida	DSM 44437	Labeda et al. 2001	validly published under the ICNP	correct name	soil	Y	Y
Lentzea albidocapillata	DSM 44073	Yassin et al. 1995	validly published under the ICNP	correct name	tissue specimen of a 46 year-old woman suffering from peritoneal carcinomatosis following carcinome of the colon	Y	Y
Lentzea atacamensis	see L. deserti.	(Okoro et al. 2010) Nouioui et al. 2018	validly published under the ICNP	correct name	Desert soil	Y	Y

Lentzea californiensis	DSM 43393	Labeda et al. 2001	validly published under the ICNP	correct name	soil	Y	Y
Lentzea cavernae	CGMCC 4.7367	Fang et al. 2017	validly published under the ICNP	correct name	soil	Y	Y
Lentzea deserti	DSM 45480	(Okoro et al. 2010) Nouioui et al. 2018	validly published under the ICNP	synonym	Desert soil	Y	Y
Lentzea flava	JCM 3296	(Gauze et al. 1974) Nouioui et al. 2018	validly published under the ICNP	correct name	soil	Y	Y
Lentzea flaviverrucosa	DSM 44664	(ex Yan and Deng 1966) Xie et al. 2002	validly published under the ICNP	correct name	soil	Y	Y
Lentzea fradiae	CGMCC 4.3506	(Zhang et al. 2007) Nouioui et al. 2018	validly published under the ICNP	correct name	soil	Y	Y
Lentzea guizhouensis	DHS C013	Cao et al. 2016	validly published under the ICNP	correct name	Karst limestone	Y	Y
"Lentzea indica"	PSKA42	Maiti and Mandal 2020	not validly published	not validly published	soil	Y	Y
Lentzea jiangxiensis	CGMCC 4.6609	Li et al. 2012	validly published under the ICNP	correct name	acidic soil	Y	Y
Lentzea kentuckyensis	NRRL B-24416	Labeda et al. 2007	validly published under the ICNP	correct name	equine placenta	Y	Y
Lentzea nigeriaca	DSM 45680	(Camas et al. 2013) Nouioui et al. 2018	validly published under the ICNP	correct name	soil	Y	Y

Lentzea pudingi	CGMCC 4.7319	Cao et al. 2017	validly published under the ICNP	correct name	soil	Y	Y
Lentzea terrae	NEAU-LZS 42	Li et al. 2018	validly published under the ICNP	correct name	soil	Y	Y
Lentzea tibetensis	FXJ1.1311	Huang and Huang 2021	validly published under the ICNP	correct name	soil	Y	Y
Lentzea violacea	DSM 44796	(Lee et al. 2000) Labeda et al. 2001	validly published under the ICNP	synonym	soil from gold mine cave	Y	Y
Lentzea waywayandensis	DSM 44232	(Labeda and Lyons 1989) Labeda et al. 2001	validly published under the ICNP	correct name	soil	Y	Y
Lentzea xinjiangensis	CGMCC 4.3525	(Wang et al. 2007) Nouioui et al. 2018	validly published under the ICNP	correct name	soil	Y	Y
Lentzea chajnantorensis		Idris et al. 2017	validly published under the ICNP	correct name	soil	N	N
"Lentzea isolaginshaensis"		Wang et al. 2019	not validly published	not validly published	soil	N	N
Lentzea rhizosphaerae		(Zhao et al. 2017) Yang and Zhi 2020	validly published under the ICNP	correct name	soil	N	N
Lentzea roselyniae		(Okoro et al. 2010) Nouioui et al. 2018	validly published under the ICNP	correct name	soil	N	N
Lentzea soli		Li et al. 2018	validly published under the ICNP	correct name	soil	N	N

**Name**	**Strain No.**	**Genome available June 2022 and used for TYGS/Database**	**Accession No.**	**Assembly type/ No. of scafolds or contigs**	**Genome size (bp)**	**N50 (bp)**	**GC content (%)**	**No. of CDSs**	**No. of RNA genes**
Lentzea aerocolonigenes	NBRC 13195	Y/ NCBI	GCF_000974445.1	Scaffold/55	10698154	364408	68.9	9898	77
Lentzea alba	NEAU-D13	Y/ NCBI	GCF_011067745.1	Scaffold/35	10211053	579.675	68.7	9232	83
Lentzea albida	DSM 44437	Y/ NCBI	GCF_900111005.1	Scaffold/40	9441135	352.714	70.1	8818	78
Lentzea albidocapillata	DSM 44073	Y/ NCBI	GCF_900176525.1	Scaffold/37	8639486	453453	68.7	8185	76
Lentzea atacamensis	see L. deserti.	Y/ NCBI	GCA_003148865.1	Scaffold/32	9306230	785641	68.9	9058	76
Lentzea californiensis	DSM 43393	Y/EzTaxon	jgi.1048875.1	Scaffold/34	8998498	528.83	69.3	8539	87
Lentzea cavernae	CGMCC 4.7367	Y/ NCBI	GCF_014653695.1	Scaffold/39	9738650	540.55	69.6	9370	68
Lentzea deserti	DSM 45480	Y/EzTaxon	GCA_003148865.1	Scaffold/41	9529573	445113	68.8	9224	76
Lentzea flava	JCM 3296	Y/ NCBI	GCF_014648475.1	Scaffold/136	9746996	141681	69	9203	84
Lentzea flaviverrucosa	DSM 44664	Y/ NCBI	GCF_003350445.1	Scaffold/31	9468454	462737	69.2	8932	78
Lentzea fradiae	CGMCC 4.3506	Y/ NCBI	GCF_900100955.1	Scaffold/43	8508028	479571	70.5	8020	75
Lentzea guizhouensis	DHS C013	Y/ NCBI	GCF_001701025.1	Complete Genome	9997872	9997872	70	9760	84
"Lentzea indica"	PSKA42	Y/ NCBI	GCF_012184385.1	Scaffold/637	9967719	29611	68.3	8900	68

Lentzea jiangxiensis	CGMCC 4.6609	Y/ NCBI	GCF_900104175.1	Scaffold/61	8591279	279756	70.2	8027	79
Lentzea kentuckyensis	NRRL B-24416	Y/ NCBI	GCF_002150765.1	Scaffold/261	10210611	99903	68.7	9374	68
Lentzea nigeriaca	DSM 45680	Y/ NCBI	GCF_016907955.1	Scaffold/42	9332327	641913	68.9	8982	71
Lentzea pudingi	CGMCC 4.7319	Y/ NCBI	GCF_014646255.1	Scaffold/65	9209069	402083	69.1	9076	71
Lentzea terrae	NEAU-LZS 42	Y/ NCBI	GCF_003265345.1	Scaffold/59	10581732	476156	68.6	9915	71
Lentzea tibetensis	FXJ1.1311	Y/ NCBI	GCF_007845675.1	Scaffold/120	9370757	199708	69.5	8919	58
Lentzea violacea	DSM 44796	Y/ NCBI	GCA_900100275.1	Scaffold/57	8671075	331077	69	8301	83
Lentzea waywayandensis	DSM 44232	Y/ NCBI	GCF_900115955.1	Scaffold/33	10153412	627394	68.9	9339	82
Lentzea xinjiangensis	CGMCC 4.3525	Y/ NCBI	GCF_900110955.1	Scaffold/54	8684108	313534	70.7	8199	76
Lentzea chajnantorensis		N							
"Lentzea isolaginshaensis"		N							
Lentzea rhizosphaerae		N							
Lentzea roselyniae		N							
Lentzea soli		N							

References:

Camas, M., Veyisoglu, A., Tatar, D., Saygin, H., Cetin, D., Sazak, A., Guven, K., and Sahin, N. "Lechevalieria nigeriaca sp. nov., isolated from arid soil." Int. J. Syst. Evol. Microbiol. (2013) 63:3750-3754.

Cao, C., Yuan, B., Qin, S., Jiang, J., Tao, F., and Lian, B. "Lentzea pudingi sp. nov., isolated from a weathered limestone sample in a karst area." Int. J. Syst. Evol. Microbiol. (2017) 67:4873-4878.

Cao, C.-L., Zhou, X.-Q., Qin, S., Tao, F.-X., Jiang, J.-H., and Lian, B. "Lentzea guizhouensis sp. nov., a novel lithophilous actinobacterium isolated from limestone from the Karst area, Guizhou, China." Antonie van Leeuwenhoek (2015) 108:1365-1372.

Fang, B.-Z., Han, M.-X., Liu, L., Zhang, Z.-T., Liu, W.-L., Shen, J.-T., Wang, Y., Zhang, W.-Q., Wei, D.-Q., and Li, W.-J. "Lentzea cavernae sp. nov., an actinobacterium isolated from a karst cave sample, and emended description of the genus Lentzea." Int. J. Syst. Evol. Microbiol. (2017) 67:2357-2362.

Gauze, G.F., Maksimova, T.S., Ollkhovatova, O.L., Sveshnikova, M.A., Kochetkova, G.V., and Ilchenko, G.B. "Production of madumycin, an antibacterial antibiotic, by Actinomadura flava sp. nov." Antibiotiki (1974) 19:771-775. [No PubMed record available.]

Huang, J., and Huang, Y. "Lentzea tibetensis sp. nov., a novel Actinobacterium with antimicrobial activity isolated from soil of the Qinghai-Tibet Plateau." Int. J. Syst. Evol. Microbiol. (2021) 71(8):004976.

Idris, H., Nouioui, I., Asenjo, J.A., Bull, A.T., and Goodfellow, M. "Lentzea chajnantorensis sp. nov., an actinobacterium from a very high altitude Cerro Chajnantor gravel soil in northern Chile." Antonie van Leeuwenhoek (2017) 110:795-802.

Labeda, D.P. "Transfer of 'Nocardia aerocolonigenes' (Shinobu andKawato 1960) Pridham 1970 into the genus Saccharothrix Labeda, Testa, Lechevalier, and Lechevalier 1984 as Saccharothrix aerocolonigenes sp. nov." Int. J. Syst. Bacteriol. (1986) 36:109-110. [No PubMed record available.]

Labeda, D.P., and Lyons, A.J. "Saccharothrix texasensis sp. nov. and Saccharothrix waywayandensis sp. nov." Int. J. Syst. Bacteriol. (1989) 39:355-358.

Labeda, D.P., Donahue, J.M., Sells, S.F., and Kroppenstedt, R.M. "Lentzea kentuckyensis sp. nov., of equine origin." Int. J. Syst. Evol. Microbiol. (2007) 57:1780-1783.

Labeda, D.P., Hatano, K., Kroppenstedt, R.M., and Tamura, T. "Revival of the genus Lentzea and proposal for Lechevalieria gen. nov." Int. J. Syst. Evol. Microbiol. (2001) 51:1045-1050.

Li, D., Jiang, H., Han, L., Li, Y., Zhao, J., Jiang, S., Wang, X., and Xiang, W. "Lentzea terrae sp. nov., isolated from soil and an emended description of Lentzea soli." Int. J. Syst. Evol. Microbiol. (2018) 68:3528-3533.

Li, D., Zheng, W., Zhao, J., Han, L., Zhao, X., Jiang, H., Wang, X., and Xiang, W. "Lentzea soli sp. nov., an actinomycete isolated from soil." Int. J. Syst. Evol. Microbiol. (2018) 68:1496-1501.

Li, X., Zhang, L., Ding, Y., Gao, Y., Ruan, J., and Huang, Y. "Lentzea jiangxiensis sp. nov., isolated from acidic soil." Int. J. Syst. Evol. Microbiol. (2012) 62:2342-2346.

Maiti, P.K., and Manda,l S. "Lentzea indica sp. nov., a novel actinobacteria isolated from Indian Himalayan-soil." Antonie van Leeuwenhoek (2020) 113(10):1411-1423.

Nouioui, I., Carro, L., García-López, M., Meier-Kolthoff, J.P., Woyke, T., Kyrpides, N.C., Pukall, R., Klenk, H.-P., Goodfellow, M., and Göker M. "Genome-based taxonomic classification of the phylum Actinobacteria." Front. Microbiol. (2018) 9:2007.

Okoro, C.K., Bull, A.T., Mutreja, A., Rong, X., Huang, Y., and Goodfellow, M. "Lechevalieria atacamensis sp. nov., Lechevalieria deserti sp. nov. and Lechevalieria roselyniae sp. nov., isolated from hyperarid soils." Int. J. Syst. Evol. Microbiol. (2010) 60(2):296-300.

Sun, X., Zhao, J., Luo, X., Hou, W., Xiang, W., Song, J., and Wang, X. "Lentzea alba sp. nov., a novel actinobacterium isolated from soil." Int. J. Syst. Evol. Microbiol. (2021) 71(2):004661.

Wang, L., Li, Y., and Li, Y. "Lentzea isolaginshaensis sp. nov., an actinomycete isolated from desert soil." Antonie van Leeuwenhoek (2019) 112(4):633-639.

Wang, W., Zhang, Z., Tang, Q., Mao, J., Wei, D., Huang, Y., Liu, Z., Shi, Y., and Goodfellow, M. "Lechevalieria xinjiangensis sp. nov., a novel actinomycete isolated from radiation-polluted soil in China." Int. J. Syst. Evol. Microbiol. (2007) 57:2819-2822.

Xie, Q., Wang, Y., Huang, Y., Wu, Y., Ba, F., and Liu, Z. "Description of Lentzea flaviverrucosa sp. nov. and transfer of the type strain of Saccharothrix aerocolonigenes subsp. staurosporea to Lentzea albida." Int. J. Syst. Evol. Microbiol. (2002) 52(5):1815-1820. Published online 5 July 2002. DOI 10.1099/ijs.0.02204-0

Yang, L.L., and Zhi, X.Y. Reclassification of Friedmanniella endophytica, Lysinimicrobium sediminis and Lechevalieria rhizosphaerae as Microlunatus kandeliicorticis nom. nov., Demequina sediminis comb. nov. and Lentzea rhizosphaerae comb. nov., respectively." Int. J. Syst. Evol. Microbiol. (2020) 70(6):3930-3931.

Yassin, A.F., Rainey, F.A., Brzezinka, H., Jahnke, K.D., Weissbrodt, H., Budzikiewicz, H., Stackebrandt, E., and Schaal, K.P. "Lentzea gen. nov., a new genus of the order Actinomycetales." Int. J. Syst. Bacteriol. (1995) 45:357-363.

Zhang, J., Xie, Q., Liu, Z., and Goodfellow, M. "Lechevalieria fradiae sp. nov., a novel actinomycete isolated from soil in China." Int. J. Syst. Evol. Microbiol. (2007) 57:832-836.

Zhao, J., Li, W., Shi, L., Wang, H., Wang, Y., Zhao, Y., Xiang, W., and Wang, X. "Lechevalieria rhizosphaerae sp. nov., a novel actinomycete isolated from rhizosphere soil of wheat (Triticum aestivum L.) and emended description of the genus Lechevalieria." Int. J. Syst. Evol. Microbiol. (2017) 67:4655-4659.

Supplementary Table 3. ANI results (%) between the BCCO strains and the reported type strains.

**Strain No.**	**BCCO 10_0061^T**	**BCCO 10_0798^T**	**BCCO 10_0856^T**
BCCO 10_0061^T	100.00	90.72	87.74
BCCO 10_0798^T	90.72	100.00	87.79
BCCO 10_0856^T	87.74	87.79	100.00
Lentzea aerocolonigenes NBRC 13195^T	86.98	87.03	87.64
Lentzea alba NEAU-D13^T	87.37	87.29	89.69
Lentzea albida DSM 44437^T	88.50	88.84	86.54
Lentzea albidocapillata subsp. *albidocapillata* DSM 44073^T	90.95	94.26	87.72
Lentzea albidocapillata subsp. *violacea* DSM 44796^T	90.80	93.99	87.63
Lentzea atacamensis DSM 45479^T	86.60	86.67	87.72
Lentzea californiensis DSM 43393^T	90.54	94.04	87.38
Lentzea cavernae CGMCC 4.7367^T	89.24	89.52	86.87
Lentzea deserti DSM 45480^T	86.62	86.65	87.65
Lentzea flava JCM 3296^T	86.42	86.53	87.30
Lentzea flaviverrucosa DSM 44664^T	90.50	94.01	87.47
Lentzea fradiae CGMCC 4.3506^T	85.65	85.88	85.38
Lentzea guizhouensis DHS C013^T	85.09	85.37	85.23
Lentzea indica PSKA42^T	87.33	87.31	88.50
Lentzea jiangxiensis CGMCC 4.6609^T	87.93	88.24	86.60
Lentzea kentuckyensis NRRL B-24416^T	87.31	87.19	89.62
Lentzea nigeriaca DSM 45680^T	86.26	86.42	87.19
Lentzea pudingi CGMCC 4.7319^T	89.57	90.56	87.02
Lentzea terrae DSM 105696^T	86.32	86.45	87.33
Lentzea tibetensis FXJ1.1311^T	80.73	80.88	80.79
Lentzea waywayandensis DSM 44232^T	94.03	90.73	87.86
Lentzea xinjiangensis CGMCC 4.3525^T	86.94	87.18	86.97

Supplementary Table 4. Results of digital DNA-DNA hybridization (%) between the BCCO strains and the reported type strains.

**Strain No.**	**BCCO 10_0061^T**	**BCCO 10_0798^T**	**BCCO 10_0856^T**
BCCO 10_0061^T	100	56.4	54.3
BCCO 10_0798^T	56.4	100	54.1
BCCO 10_0856^T	54.3	54.1	100
Lentzea aerocolonigenes NBRC 13195^T	43	44.4	45.9
Lentzea alba NEAU-D13^T	44.8	46.4	51.4
Lentzea albida DSM 44437^T	53.8	51.2	46.5
Lentzea albidocapillata subsp. *albidocapillata* DSM 44073^T	53.7	59.9	49.1
Lentzea albidocapillata subsp. *violacea* DSM 44796^T	49.8	59.5	47.1
Lentzea atacamensis DSM 45479^T	44.5	46.8	49.2
Lentzea californiensis DSM 43393^T	56.1	66.2	49.5
Lentzea cavernae CGMCC 4.7367^T	51.9	51.2	45
Lentzea deserti DSM 45480^T	43.9	47.1	48
Lentzea flava JCM 3296^T	45.2	44.7	50.1
Lentzea flaviverrucosa DSM 44664^T	56.5	64	50.3
Lentzea fradiae CGMCC 4.3506^T	40.7	41.6	38.6
Lentzea guizhouensis DHS C013^T	41.1	41	40.2
Lentzea indica PSKA42^T	46.5	46	52.9
Lentzea jiangxiensis CGMCC 4.6609^T	43.9	46.9	41.2
Lentzea kentuckyensis NRRL B-24416^T	44.3	44.4	51.6
Lentzea nigeriaca DSM 45680^T	43.6	42.3	46.6
Lentzea pudingi CGMCC 4.7319^T	50.6	55.9	47.5
Lentzea terrae DSM 105696^T	44.6	43.9	49.2
Lentzea tibetensis FXJ1.1311^T	24.4	25.1	25.3
Lentzea waywayandensis DSM 44232^T	67.7	55.2	55.2
Lentzea xinjiangensis CGMCC 4.3525^T	44.1	43.8	43.4

Supplementary Table 5. List of the 100 best scored gene families used for the Core genome multi-locus sequence typing.

PGFam	Alignment Score	Aligment Length	Mean Square Frequencies	Product
PGF_00045963	31.47	1125	938	Pyruvate carboxylase (EC 6.4.1.1)
PGF_08152874	30.9	1322	0.85	ATP-dependent helicase HrpA
PGF_10049811	26.82	1033	835	Protein translocase subunit SecA
PGF_02975845	26.31	1028	821	putative membrane protein
PGF_00010349	26.15	770	942	Guanosine-3',5'-bis(diphosphate) 3'-pyrophosphohydrolase (EC 3.1.7.2) / GTP pyrophosphokinase (EC 2.7.6.5), (p)ppGpp synthetase II
PGF_00009968	25.62	1175	748	Glycyl-tRNA synthetase alpha chain (EC 6.1.1.14) / Glycyl-tRNA synthetase beta chain (EC 6.1.1.14)
PGF_03104485	25.61	765	926	Polyribonucleotide nucleotidyltransferase (EC 2.7.7.8)
PGF_00705001	24.61	707	926	Enoyl-CoA hydratase (EC 4.2.1.17) / 3-hydroxyacyl-CoA dehydrogenase (EC 1.1.1.35) / 3-hydroxybutyryl-CoA epimerase (EC 5.1.2.3)
PGF_10370701	24.1	778	864	Xanthine dehydrogenase, molybdenum binding subunit (EC 1.17.1.4)
PGF_00021022	23.8	764	861	Methylmalonyl-CoA mutase large subunit, MutB (EC 5.4.99.2)
PGF_00071558	23.35	600	953	Bacterial proteasome-activating AAA-ATPase (PAN)
PGF_01175575	23.02	695	873	Threonyl-tRNA synthetase (EC 6.1.1.3)
PGF_04674543	22.03	555	935	DNA repair helicase
PGF_00421679	21.96	651	861	DNA primase DnaG
PGF_00036330	21.38	570	896	Predicted sodium-dependent galactose transporter
PGF_00810218	21.18	804	747	Na(+)-dependent bicarbonate transporter BicA / Carbonic anhydrase, beta class (EC 4.2.1.1)
PGF_00009966	20.46	473	941	Glycyl-tRNA synthetase (EC 6.1.1.14)
PGF_06447349	20.23	473	0.93	NADP-dependent malic enzyme (EC 1.1.1.40)
PGF_00024983	20.07	650	787	NHL repeat containing protein
PGF_08562657	19.37	533	839	tRNA-i(6)A37 methylthiotransferase (EC 2.8.4.3)
PGF_00423429	19.3	574	806	Dihydroxyacetone kinase-like protein, phosphatase domain / Dihydroxyacetone kinase-like protein, kinase domain

PGF_00051220	18.97	536	819	Serine phosphatase RsbU, regulator of sigma subunit
PGF_05572316	18.96	469	875	FIG007959: peptidase, M16 family
PGF_03035890	18.86	403	939	Methylsuccinyl-CoA dehydrogenase, predicted by (Erb et al, 2007)
PGF_00048846	18.79	484	854	Ribosomal protein S12p Asp88 (E. coli) methylthiotransferase (EC 2.8.4.4)
PGF_07760799	18.63	554	791	Pyruvate kinase (EC 2.7.1.40)
PGF_05554840	18.38	545	787	Cobyric acid synthase (EC 6.3.5.10)
PGF_00010332	18.24	461	849	Guanine deaminase (EC 3.5.4.3)
PGF_00024479	18.14	400	907	NAD-dependent malic enzyme (EC 1.1.1.38)
PGF_00020562	18.11	522	793	Metallopeptidase
PGF_10555225	18.1	421	882	Ornithine aminotransferase (EC 2.6.1.13)
PGF_00420975	17.98	423	874	D-inositol-3-phosphate glycosyltransferase (EC 2.4.1.250)
PGF_00063916	17.97	447	0.85	Tyrosyl-tRNA synthetase (EC 6.1.1.1)
PGF_06759833	17.85	370	928	Peptide chain release factor 2
PGF_00418439	17.68	461	823	Coenzyme F420-0:L-glutamate ligase (EC 6.3.2.31) @ F420-1:L-glutamate ligase (EC 6.3.2.34) / Nitroreductase family protein Rcas_3978
PGF_06755829	17.66	402	881	DNA polymerase III delta prime subunit (EC 2.7.7.7)
PGF_02407790	17.62	432	848	3-ketoacyl-CoA thiolase (EC 2.3.1.16) @ Acetyl-CoA acetyltransferase (EC 2.3.1.9), FadA2
PGF_00007012	17.36	382	888	GTP-binding and nucleic acid-binding protein YchF
PGF_01400330	17.35	346	933	Biotin synthase (EC 2.8.1.6)
PGF_00024274	17.32	407	858	N5-carboxyaminoimidazole ribonucleotide synthase (EC 6.3.4.18)
PGF_07015581	17.19	387	874	Carbamoyl-phosphate synthase small chain (EC 6.3.5.5)
PGF_00002883	17.01	422	828	Uncharacterized protein Rv3311
PGF_09129813	16.99	408	841	Glutamate 5-kinase (EC 2.7.2.11) / RNA-binding C-terminal domain PUA
PGF_03520151	16.92	556	717	Cell division protein FtsZ
PGF_06671647	16.89	401	843	N-formylglutamate deformylase (EC 3.5.1.68) [alternative form]
PGF_00016074	16.84	417	825	L-cysteine:1D-myo-inosityl 2-amino-2-deoxy-alpha-D-glucopyranoside ligase MshC (EC 6.3.1.13)
PGF_01867628	16.73	351	893	Heat-inducible transcription repressor HrcA

PGF_00024267	16.7	332	916	N5,N10-methylenetetrahydromethanopterin reductase-related protein, SCO6416-type
PGF_01206271	16.59	454	779	probable L-gulonolactone oxidase(EC:1.1.3.-)
PGF_07063065	16.58	340	899	Transcription termination protein NusA
PGF_00008611	16.57	336	904	Glycerol-3-phosphate dehydrogenase [NAD(P)+] (EC 1.1.1.94)
PGF_00402161	16.54	610	0.67	Beta-lactamase class C-like protein in cluster with YzbB
PGF_03750541	16.33	364	856	Aminopeptidase YpdF (MP-, MA-, MS-, AP-, NP- specific)
PGF_04068298	16.25	328	897	Methyltransferase Rv3038c, type 11
PGF_00963535	16.25	608	659	Lipoprotein LpqB, modulates activity of MtrAB two-component system
PGF_03122412	16.07	479	734	Arginyl aminopeptidase (EC 3.4.11.6) @ Leucyl aminopeptidase (EC 3.4.11.10)
PGF_00021023	16	855	547	Methylmalonyl-CoA mutase small subunit, MutA (EC 5.4.99.2)
PGF_00015357	15.9	543	682	Iron(II)-dependent oxidoreductase EgtB (hercynine sythase)
PGF_01683273	15.9	419	777	Glutamate--cysteine ligase (EC 6.3.2.2)
PGF_03102190	15.78	475	724	PTS system, N-acetylglucosamine-specific IIC component
PGF_04746167	15.68	1191	454	uncharacterized protein with VanW-like domain
PGF_08316603	15.64	463	727	Methionine gamma-lyase (EC 4.4.1.11)
PGF_02390924	15.5	325	0.86	16S rRNA (cytosine(1402)-N(4))-methyltransferase (EC 2.1.1.199)
PGF_00064043	15.49	311	878	UDP-galactofuranosyl transferase GlfT1 (EC 2.4.1.287), catalyzes initiation of cell wall galactan polymerization
PGF_04010787	15.36	331	844	Uncharacterized metalohydrolase SCO3582
PGF_03475877	15.34	344	827	Chromosome (plasmid) partitioning protein ParB
PGF_00416795	15.29	397	767	3,5-diaminohexanoate dehydrogenase (EC 1.4.1.11)
PGF_04835795	15.28	328	844	Site-specific tyrosine recombinase XerC
PGF_00421758	15.05	352	802	DNA recombination protein RmuC
PGF_01476110	15.04	361	792	YgfD: protein that forms a complex with the methylmalonyl-CoA mutase in a pathway for conversion of succinyl-CoA to propionyl-CoA
PGF_00053671	14.95	296	869	Spermidine synthase (EC 2.5.1.16)
PGF_05579323	14.64	354	778	L-threonine 3-O-phosphate decarboxylase (EC 4.1.1.81)
PGF_00425295	14.64	403	729	Extracellular ribonuclease Bsn
PGF_00016284	14.62	262	903	LOG family protein
PGF_02923127	14.54	261	0.9	Uridylate kinase (EC 2.7.4.22)

PGF_03790040	14.47	276	871	Ribonuclease III (EC 3.1.26.3)
PGF_00019593	14.42	315	813	Membrane protease family protein BA0301
PGF_00624692	14.32	431	0.69	Glucose/sorbosone dehydrogenase, lipoprotein LppZ
PGF_00038986	14.28	283	849	Purine nucleoside phosphorylase (EC 2.4.2.1)
PGF_10348836	14.26	294	832	NADH-ubiquinone oxidoreductase chain J (EC 1.6.5.3)
PGF_07609122	14.26	288	0.84	Pyrroline-5-carboxylate reductase (EC 1.5.1.2)
PGF_00064393	14.22	301	0.82	UTP--glucose-1-phosphate uridylyltransferase (EC 2.7.7.9)
PGF_04211832	14.09	268	861	Polyphosphate glucokinase (EC 2.7.1.63)
PGF_00413208	14.09	255	882	tRNA (guanine(37)-N(1))-methyltransferase (EC 2.1.1.228)
PGF_00000584	13.96	264	859	Uncharacterized protien SCO1664
PGF_10414515	13.91	343	751	Nucleoside triphosphate pyrophosphohydrolase MazG (EC 3.6.1.8)
PGF_02450432	13.89	301	0.8	Phosphatidate cytidylyltransferase (EC 2.7.7.41)
PGF_00041289	13.87	258	863	Putative hemolysin
PGF_02472178	13.74	281	0.82	Uncharacterized metal-dependent hydrolase YcfH
PGF_00049893	13.74	201	969	SSU ribosomal protein S4p (S9e), zinc-independent
PGF_00876943	13.72	301	791	1,4-dihydroxy-2-naphthoate polyprenyltransferase (EC 2.5.1.74)
PGF_00410417	13.69	394	0.69	Putative secreted lipase
PGF_00016431	13.68	216	931	LSU ribosomal protein L3p (L3e)
PGF_00047155	13.64	282	812	Redox-sensing transcriptional repressor Rex
PGF_06274006	13.53	283	804	ATP synthase delta chain (EC 3.6.3.14)
PGF_00943546	13.52	283	804	Methylglutaconyl-CoA hydratase (EC 4.2.1.18)
PGF_00012356	13.47	311	764	Homoserine kinase (EC 2.7.1.39)
PGF_03952305	13.45	378	692	Arogenate dehydrogenase (EC 1.3.1.43)
PGF_00406542	13.33	262	824	FIG00814129: Possible chaperone
PGF_03174068	13.31	316	749	Transcription antitermination protein NusG

Supplementary Table 6. Biosynthetic gene clusters indentified and the sequence similarity (%) in the genomes of BCCO strains using antiSMASH.

**Most similar gene cluster**	**class**	**BCCO 10_0061ᵀ**	**BCCO 10_0798ᵀ**	**BCCO 10_0856ᵀ**
Geosmin	terpene	100	100(2x)	100(2x)
Nystatin A1	PKS type I	78	0	72
Ery-9 / Ery-6 / Ery-8 / Ery-7 / Ery-5 / Ery-4 / Ery-3 (erythromycin)	RiPP:Lanthipeptide	75	75	75
coelichelin	NRP	72	72	72
isorenieratene	terpene	85	85	0
epsilon Poly-L-lysine	NAPPA	100	100	100
2-methylisoborneol	terpene	50	50	0
iso-migrastatin / migrastatin / dorrigocin A / dorrigocin B / 13-epi-dorrigocin A	PKS type I	54	0	0
indigoidine	NRP	60	60	0
staurosporine	alkaloid	0	80	0
minimycin	NRP+saccharide	60	60	0

Supplementary Table 7. Complete fatty acid profiles of BCCO strains.

**Fatty acids**	**BCCO 10_0061^T (%)**	**BCCO 10_0798^T (%)**	**BCCO 10_0856^T (%)**
iso-$C_{12:0}$	0.20	-	0.13
$C_{12:0}$	0.17	0.18	0.11
iso-$C_{13:0}$	0.21	0.13	0.18
anteiso-$C_{13:0}$	0.24	0.15	0.18
$C_{13:0}$	0.17	0.09	0.13
iso-$C_{14:0}$	4.22	2.39	3.66
$C_{14:0}$	1.87	3.71	2.45

iso-$C_{15:1}$ ω5c	0.10	-	-
iso-$C_{15:0}$	13.24	10.20	12.94
anteiso-$C_{15:0}$	18.91	15.53	18.40
$C_{15:1}$ ω6c	1.11	0.49	0.72
$C_{15:0}$	3.75	1.91	3.94
iso-$C_{16:1}$ ω6c	0.91	0.45	0.41
iso-$C_{16:0}$	22.42	11.76	18.71
$C_{16:1}$ ω7c	5.00	17.73	8.96
$C_{16:0}$	10.25	20.20	13.63
iso-$C_{17:1}$ ω7c	0.50	0.30	0.40
$C_{16:0}$ 10-methyl	0.40	1.70	0.70
anteiso-$C_{17:1}$ ω7c	0.43	0.60	0.33
iso-$C_{17:0}$	1.50	1.31	1.69
anteiso-$C_{17:0}$	8.78	8.28	7.56
$C_{17:1}$ ω8c	1.83	1.03	1.56
$C_{17:1}$ ω6c	0.24	0.29	0.26
$C_{17:0}$	2.39	1.29	2.62
iso-$C_{18:0}$	0.13	-	0.11
$C_{18:2}$ ω6,9c	0.28	0.23	0.29
$C_{18:1}$ ω9c	0.27	0.72	0.37
$C_{18:1}$ ω7c	-	0.57	0.15
$C_{18:0}$	0.50	1.08	0.69

Supplementary Table 8. Enzyme activity of BCCO strains detected in API ZYM assay.

**Enzyme**	**BCCO 10_0061^{T}**	**BCCO 10_0798^{T}**	**BCCO 10_0856^{T}**
Alkaline phosphalase	+	+	+
Esterase	+	+	+
Esterase Lipase	+	+	+
Lipase	W	+	-
Leucine arylamidase	+	+	+
Valine arylamidase	+	+	+
Cystine arylamidase	+	+	+
Trypsin	+	+	+
α-chymotrypsin	+	+	+
Acid phosphatase	+	+	+
Naphthol-AS-BI-phosphohydrolase	+	+	+
α-galactosidase	+	+	+
ß-galactosidase	+	+	+
ß-glucuronidase	-	W	+
α-glucosidase	+	+	+
ß-glucosidase	+	+	+
N-acetyl-ß-glucosaminidase	+	+	+
alfa-naphthyl-N-acetyl-ßD-glucosaminide	+	+	+
α-fucosidase	-	-	W

Abbreviation: W - weak activity, + positive activity, - negative activity.
